# Evaluation and driving factors of land use economic efficiency in China's urban agglomerations under the impact of COVID-19 epidemic

**DOI:** 10.3389/fpubh.2022.1016701

**Published:** 2022-09-23

**Authors:** Jianhua Wang, Junwei Ma

**Affiliations:** Business School, Changshu Institute of Technology, Suzhou, China

**Keywords:** land use economic efficiency, land market, efficiency evaluation, driving factors, urban agglomeration

## Abstract

Land is an indispensable factor of production and the basic support for all social and economic activities. The COVID-19 epidemic has a great impact on China's macro-economy and land market. As a unit with a high concentration of economic entities, urban agglomeration is closely related to its land use economic efficiency. Under the impact of epidemic and the rigid constraints of the relative scarcity of land resources, improving the land use economic efficiency is crucial to the sustainable development of urban agglomerations. Taking the 10 major urban agglomerations in China as a case study, this paper constructs a theoretical and empirical analysis framework for the land use economic efficiency and its driving mechanism of urban agglomerations, and measures the land use economic efficiency of urban agglomerations from the aspects of single factor productivity and total factor productivity. The results show that the COVID-19 epidemic has a great impact on the land market of various cities in China's urban agglomerations. Whether single factor productivity or total factor productivity is used to measure land use economic efficiency of urban agglomerations, the driving effects of industrial agglomeration, industrial structure change, technological progress, and transportation infrastructure are all significant. It is necessary to take a series of measures to reform the market-oriented allocation of land elements, and improve a long-term mechanism for the smooth operation of the land market. It is necessary to improve the land use economic efficiency through a combination of industrial agglomeration, industrial structure adjustment, technological progress, and transportation infrastructure.

## Introduction

Over the past few decades, urbanization and industrialization have become a global phenomenon. Especially in China, the speed of urbanization and industrialization is unprecedented and has attracted global attention. In the process of rapid urbanization and industrialization, cities are no longer isolated, but rapidly concentrated and linked together in the form of urban agglomerations. In the world of globalization and information explosion, the high-quality development of urban agglomerations is of increasing importance. Facing the challenges of globalization, urban agglomerations actively participate in global urban competition, division and cooperation by virtue of industrial clusters and scale economies, and are also playing an increasingly important role in international competition. In particular, some world-class urban agglomerations are not only the core of national economic development, but also extend the administrative boundaries, profoundly affecting the world's economic and social development. In fact, urban agglomerations have become technology incubation centers, production factor allocation centers and wealth creation centers, and are becoming more and more important. Under the influence of market mechanism and government regulation, various factors such as talent, technology, knowledge, information, and capital are continuously concentrated in competitive urban agglomerations (1–3). Urban agglomerations have become an important strategic approach for countries to develop productivity and optimize production factors.

With the rapid development of urbanization, the land use efficiency of urban has increasingly become a key factor affecting economic, social and environmental development. Land is an indispensable factor of production and the basic support for all social and economic activities. As a unit with a high concentration of economic entities, urban agglomeration is closely related to the utilization of land resources. Land is the spatial carrier of social and economic development, and rational and efficient utilization of land resources is an important guarantee for economic stability and sustainable growth (4). Under the rigid constraints of the relative scarcity of land resources, the contradiction between urban land use and the quality of economic growth has become increasingly prominent. The development model of driving the regional economy by expanding the scale of the city is unsustainable (5). Therefore, promoting the cooperation of various types of urban land, thereby improving the land use economic efficiency, has become an important path to promote the sustainable development of urban agglomerations. Land Use Economic Efficiency (LUEE) of urban agglomerations is a manifestation of the realization degree of land value in economic development, and an intuitive reflection of the economic efficiency of industrial activities. LUEE in urban agglomerations reflects the ratio of input and output of land resources, including two interrelated levels. This paper measures LUEE from the perspectives of single factor productivity and total factor productivity. LUEE in urban agglomerations is an important driving force for the improvement of the quality of economic growth. Therefore, under the rigid constraints of limited urban land supply, improving LUEE is also an important way to solve the problem of excessive land cost in the economic growth of urban agglomerations.

At present, urban agglomeration is still in the stage of rapid development. With the continuous improvement of the economic level, the population of urban agglomerations has doubled, the process of urbanization has continued to accelerate, and the demand for land has continued to increase, and the way and structure of land use will inevitably change. However, the demand for land corresponding to the rapid economic development has expanded rapidly, and the extensive land use has become increasingly prominent, which in turn will have a huge negative impact on the economic development of urban agglomerations. Different from previous research, there are four aspects of contribution: First, the coupling coefficient model is established to measure the coordination between the severity of the epidemic and the degree of impact on the land market. Second, a theoretical and empirical analysis framework for driving factors of land use economic efficiency of urban agglomerations is constructed. This paper regards land as an important economic factor, and mainly measures the land use economic efficiency of urban agglomerations from an economic point of view and analyzes the driving factors. Third, this paper constructs a comprehensive evaluation index system for land use economic efficiency of urban agglomerations composed of single factor productivity and total factor productivity. It comprehensively evaluates the land use economic efficiency of urban agglomerations and their respective driving factors can be analyzed and compared. Fourth, this paper selects multiple urban agglomerations as the case study and focus on the driving factors of land use economic efficiency. The sustainable development of urban agglomerations is closely related to the land use efficiency. As a regional economic growth pole, urban agglomeration is the gathering place of various resources. An in-depth study of land use economic efficiency of major urban agglomerations in China and its driving factors will help to clarify the average level and gap of land use economic efficiency of each city within the urban agglomeration, which is helpful to adjust human production and living activities, and improve the overall land use economic efficiency of urban agglomerations. This will promote urban agglomerations to enter the track of land intensification and high-quality economic development, and also provides relevant theoretical basis for promoting sustainable and green economic development of urban agglomerations.

The next section of this six-section paper reviews the literature and develops research hypotheses. The four section describes materials and empirical methods. The five section interprets the results. The final section summaries the major findings, contributions, followed by suggestions for future work.

## Literature review

Urban land use efficiency is always the core proposition of land science research. Scholars conducted research on intensive land use, measurement and evaluation of land use efficiency, spatial and temporal differences and evolution patterns of land use efficiency and its influencing factors, and provided many suggestions for the improvement of land use efficiency.

Scholars used mathematical statistics to measure the land use efficiency. On the basis of fully understanding the connotation of land intensification, Fonseca (6) selected indicators such as building density and plot ratio to measure urban land use efficiency (6). Harrison and Haklay (7) studied the reality and efficiency of urban land use, and established an index evaluation system and model for land use efficiency (7). Siciliano (8) integrated urbanization strategies, rural development and land use into a holistic framework for a comprehensive analysis (8). Erb et al. (9) integrated the three levels of input intensity, output intensity, and related systemic impacts of terrestrial production, and proposed a conceptual framework to quantify and analyze urban land use efficiency (9). Haque and Asam (10) established an optimization model of land use allocation in urban development zones based on genetic algorithm (10). Enrique and Vanessa (11) used mobile social media applications as an important sensor of urban land use, and used Twitter to test the land use of Manhattan, London and Madrid, and found that Twitter's positioning information could be used as a powerful data source of urban land use planning application (11). Kishii (12) established a model to evaluate the state of land use efficiency in Japanese cities, and pointed out an integrated approach to planning and utilizing public areas and private property spaces (12). Alexander et al. (13) introduced an explicit indicator and land use management support system to assess land use efficiency at the landscape level by combining land use ecosystem service indicators with land use performance indicators based of optimal land allocation, which provided certain data support for spatial planning and resource management (13).

Scholars studied the driving factors of urban land use efficiency from multiple dimensions. Daniel (14) studied the issue of urban land intensive use and believed that the improvement of urban land use efficiency was inseparable from the land intensive use and the continuous optimization of land use structure (14). Keller (15) used the FDI technology spillover effect theory to study the impact of the similarities and differences of location factors on the urban land use efficiency. Sivam (16) conducted an in-depth study of land use efficiency from an economic perspective. Tanrivermis (17) found that the main factors causing changes in land use types were agricultural productivity, population and urbanization (17). Kironde (18) studied the land use efficiency of five representative cities in Tanzania by establishing an urban land efficiency evaluation model, and proposed that the government's strong support for the urban land market could improve the efficiency and level of urban land management (18). Lambin et al. (19) pointed out that economic globalization accelerated the conversion of agricultural land and forested to urban land, and land use change posed challenges to sustainable development. Therefore, land must be regarded as an open system, using globalization to improve land use efficiency and control the irrational expansion of urban land (19). Marco et al. (20) found that the non-linear trend of urban land use efficiency in Attica, Greece, just mapped the urban expansion characteristics of the region at different stages from 1960 to 2010, and pointed out that urban land use efficiency was an important indicator of urban future growth patterns (20). Fetzel et al. (21) empirically studied the relationship between land use and yield in Africa from 1980 to 2005 and showed that land expansion could not increase yield (21). Dadi et al. (22) used GIS to explore the driving factors of urban expansion and its impact on land use change in central Ethiopia. The study showed that industrialization, residential expansion and infrastructure development were the main driving factors of land use conversion, but the transformation of land use efficiency was not high (22). Guastella et al. (23) took the Lombardy region of Italy as the research object, and constructed an economic model to analyze the decisive factors of urban spatial expansion in this region. The study showed that the larger the scale of the city, the higher the land use efficiency, and the smaller city's land occupation. The problem was less concerned and the land use efficiency was low. Therefore, effective differentiated land planning policies should be adopted to address land use issues (23). Choi and Wang (24) measured the land use efficiency of 16 cities in South Korea by using the SSBM model with undesired outputs. The study showed that the urban land use efficiency in South Korea was on the rise from 2006 to 2013, and the economic development policy of the South Korean government was an important factor in urban land use efficiency (24). Deilmann et al. (25) evaluated the land use efficiency of German cities through data envelopment analysis, and the results showed that medium-sized cities, as the development centers of residential areas and transportation areas, had the highest land use efficiency, and residential area was not the only factor determining high efficiency, high density alone did not guarantee efficiency (25).

The impact of COVID-19 and the pandemic on public spaces has been extensively assessed in public health. Scholars have pointed out strategies for responding to urban epidemics by developing a pandemic-resistant urban form at different stages of response (26). The flexibility of urban spaces has been greatly affected by COVID-19 and needs to adapt to existing structures such as public institutions or infrastructures (27). In response to the crisis, planning for an equitable and sustainable environment for citizens, economies and communities makes sense, and it is the “new normal” in urban areas (28). The risk of COVID-19 transmission is associated with land use mix, but the conclusions are inconsistent (29–31).

Judging from the existing literature, scholars have mainly analyzed the driving factors of urban land use efficiency from the aspects of location, city size, industrialization, government policies, and infrastructure. The theoretical analysis and empirical investigation of land use economic efficiency and its driving factors from the perspective of economics are rare, and it is urgent to strengthen research in this area.

## Research hypotheses

The COVID-19 epidemic in late 2019 had a huge impact on the economies of China and other countries around the world (32). Since 2022, a new round of COVID-19 epidemic has broken out in many places. Compared with the COVID-19 epidemic that occurred in 2020, this epidemic has spread faster, has more confirmed cases, and is expected to last for a longer time. This has a great impact on China's macro-economy and land market. Based on this, this paper proposes a hypothesis.

**H1: The COVID-19 epidemic has a great impact on the land market of urban agglomerations in China, and it shows great differences**.

As a special economic factor, the input-output efficiency of land is also closely related to economic agglomeration, industrial structure and other factors. Therefore, based on theory and literature analysis, this paper examines the driving factors of land use economic efficiency of urban agglomerations in China from the aspects of industrial agglomeration, industrial structure, technological progress, marketization institution and infrastructure.

Agglomeration is an inevitable trend of economic development, and urban agglomeration is the main place and important carrier of economic agglomeration. With the improvement of economic development and urbanization level, economic agglomeration is mainly manifested in the gradual improvement of the information transmission system and service system of a specific industry or related industry, which provides a basis for the realization of service sharing, infrastructure sharing and forward-backward linkage between industries. Therefore, land users in the agglomeration area can easily obtain market information and various related services, which greatly reduces transaction costs. At the same time, agglomeration will further strengthen industrial division and specialization. In this process, the land users in the agglomeration area also gradually achieve the optimal level of economic scale, and can gain the advantage of scale economy, which will greatly increase the land income. Industrial agglomeration also produces knowledge and technology spillovers. The geographical proximity of land users will promote the generation of knowledge and technology spillover effects, which is beneficial to the improvement of land use benefits. Based on this, the hypothesis is proposed as follows.

**H2: Industrial agglomeration is conducive to improving land use economic efficiency of urban agglomerations**.

The process of urban economic development is not only manifested in the growth of the total economic volume, but also in the upgrading and transformation of the industrial structure. The evolution of the industrial structure shows a certain regularity: with the acceleration of urbanization and industrialization, the primary industry is gradually transformed into the secondary industry and the tertiary industry, and convert in turn to the stage where labor-intensive industries dominate and capital-intensive industries dominate and technology-intensive industries dominate. Industrial development should rely on land, and the qualitative transformation of industrial structure needs to be reflected through changes in land use. That is to say, the evolution of industrial structure will eventually lead to changes in land use structure. Therefore, differences in industrial structure will lead to differences in land use structure and land use methods, and have different effects on the use efficiency of land resource. Based on this, the hypothesis is proposed as follows.

**H3: Industrial structure change is conducive to improving land use economic efficiency of urban agglomerations**.

Technological progress is the source of economic growth and the key to improving the economic efficiency of urban agglomerations. As the size of the metropolitan area expands, the proportion of innovators increases, making it easier to promote productivity (33). A network of cities or urban agglomeration with closely connected cities has greater diversity and creativity than individual cities, more freedom of location and less congestion (34). Batten (35) pointed out that the network city was a creative urban agglomeration in the 21st century, which had better superiority in technological cooperation innovation, so that cities in the urban agglomeration would benefit from technical exchanges and technology spillovers between cities (35). For example, European urban agglomerations are the “engine” of the European economy, and the growth of urban agglomeration economies is the result of the concentration of research-intensive and knowledge-intensive industries (36). Therefore, the technological progress of individual cities within the urban agglomeration will bring the technological progress of the urban agglomeration, and the proximity of the cities within the urban agglomeration is very conducive to mutual technical cooperation and technology spillover, which will further accelerate the technological progress of the urban agglomeration, and thus promote the land use economic efficiency of urban agglomerations. Technological progress can be measured by the level of technological innovation and technological cooperation between cities in the urban agglomeration. Based on this, the hypotheses are proposed as follows.

**H4: Technological innovation is conducive to improving land use economic efficiency of urban agglomerations**.
  **H5: Technological cooperation is conducive to improving land use economic efficiency of urban agglomerations**.

Urban agglomerations are the product of social, economic and spatial organization changes, and therefore must be influenced by institutions or policies. Urban agglomerations occur in the institutional network of market economy and government, and the minimum transaction cost is the main driving force (37). Scholars believe that the marketization mechanism and the government policy system of urban agglomeration reflect institutional changes, and the advantages or convenience brought by various institutions and policies will ultimately have an important impact on land use economic efficiency of urban agglomerations. However, the marketization institution itself is a “double-edged sword.” Based on this, the hypothesis is proposed as follows.

**H6: Marketization institution is conducive to improving land use economic efficiency of urban agglomerations**.

Urban agglomerations are gradually formed as the links between cities become increasingly close. The transportation and information infrastructure are the basis and driving force for the formation and evolution of urban agglomerations, which constitute the channel of communication between cities. Good transport infrastructure network conditions in urban agglomerations are an important factor in promoting the expansion of economic activity between cities in the region (38). The improvement of public transport in urban agglomerations can improve the availability of labor, increase information exchange and promote industrial division and professional development, so that the urban agglomeration can obtain economic benefits (39, 40). Of course, the radiation effect of transportation infrastructure in urban agglomerations is also conducive to the improvement of land use economic efficiency. Infrastructure can be measured from two main perspectives, namely, the density of transportation infrastructure and the level of information infrastructure. Based on this, the hypotheses are proposed as follows.

**H7: Transportation infrastructure is conducive to improving land use economic efficiency of urban agglomerations**.
**H8: Information infrastructure is conducive to improving land use economic efficiency of urban agglomerations**.

## Materials and methods

### Study area

This paper selects 10 urban agglomerations in China as study samples, such as the Beijing-Tianjin-Hebei, the Yangtze River Delta, the Pearl River Delta, the Shandong Peninsula, West Coast of the Straits, South Central of Liaoning, Central Plains, the middle reaches of the Yangtze River, Chengdu-Chongqing, and the Central Shanxi Plain urban agglomerations, which include a total of 122 cities. These urban agglomerations are the most fundamental areas supporting China's land development and also play a vital role in China's participation in global competition. Geographically, these 10 urban agglomerations involve three regions in the east, middle and west of China with gradient differences, and can better represent the economic development level and characteristics of the three regions in China.

### Data sources

The time range of indicator data involved in this paper is 2001–2020. Most statistical data were derived from the authoritative statistical yearbooks, including the 2002–2021 China Urban Statistical Yearbook, the 2002–2021 China Statistical Yearbook on Science and Technology, and the 2002–2021 China Statistical Yearbook. The marketization index of urban agglomerations is derived from the China Marketization Index Report (41–43). The marketization index for 2019–2020 is the forecast data.

### Methods

#### The impact of the severity of the epidemic on the land market of urban agglomerations– coupling coefficient model

In order to distinguish the difference in the impact of the severity of the epidemic on the land market in different urban agglomerations in more detail, this study uses a coupling coefficient model to measure the coordination between the severity of the epidemic and the degree of impact on the land market (44). The function expression is as follows.

(1) $$\begin{array}{l} H_{t}=\sqrt[{2}]{\frac{F_{1}\left(t,x\right)F_{2}\left(t,y\right)}{{\left[F_{1}\left(t,x\right){+F}_{2}\left(t,y\right)\right]}^{2}}} \end{array}$$

Where *H*_*t*_ is the coupling index of the COVID-19 epidemic and land market of cities in urban agglomerations at time t; *F*_1_(*t, x*) is the comprehensive evaluation value of the severity of the COVID-19 epidemic at time t (indicated by the number of confirmed cases in one million people); *F*_2_(*t, y*) is the comprehensive evaluation value of the degree of impact on the land market of cities in urban agglomerations at time t (indicated by the growth rate of comprehensive land prices). Dispersion standardization is performed on the original data of *H*_*t*_, and the result falls into the [0, 1] interval through linear transformation of the original data.

According to the coupling index, the type of coordination between the severity of the epidemic and the degree of impact on the land market of cities in urban agglomerations can be classified (Table 1). When *H*_*t*_ = 1, the coupling index is the largest, indicating that the two systems are fully coordinated at time t, and the severity of the epidemic is completely consistent with the degree of impact on the land market of cities in urban agglomerations. When 0.5 ≤ *H*_*t*_ < 1, the coupling index is large, indicating that the two systems are more coordinated at time t, and the severity of the epidemic and the degree of impact on the land market of cities in urban agglomerations is “Synchronous type.” When 0 ≤ *H*_*t*_ < 0.5, the coupling index is small, and the sensitivity of land market of cities in urban agglomerations to the COVID-19 epidemic can be judged according to the magnitudes of *F*_1_(*t, x*) and *F*_2_(*t, y*). If *F*_1_(*t, x*) > *F*_2_(*t, y*), it means that the severe epidemic does not have a great impact on the land market of cities in urban agglomerations, and the relationship between the two systems can be classified as “strong type.” If *F*_1_(*t, x*) < *F*_2_(*t, y*), it means that the mild epidemic has a greater impact on the land market of cities in urban agglomerations, and the relationship between the two systems can be classified as “fragile type.”

**Table 1:** Division of coordination type.

***H*_*t*_**	***F*_1_(*t, x*) > *F*_2_(*t, y*)**	***F*_1_(*t, x*) < *F*_2_(*t, y*)**
*H*_*t*_ = 1	fully consistent type	
0.5 ≤ *H*_*t*_ < 1	Synchronous type	
0 ≤ *H*_*t*_ < 0.5	strong type	fragile type

#### Driving factors of land use economic efficiency of urban agglomerations–panel data model

The land use economic efficiency of urban agglomerations is the performance of the realization degree of land value in economic development, and it is an intuitive reflection of the economic efficiency of industrial activities. As a measure, it is the ratio of output to input. The land use economic efficiency of urban agglomerations can be measured not only by single factor productivity, but also by total factor productivity. In essence, the land use economic efficiency of urban agglomerations is also achieved through a series of micro-mechanisms and approaches. Based on literature and theoretical analysis, this paper argues that the comprehensive effects of industrial agglomeration, industrial structure, technological progress, marketization institution and infrastructure jointly explain the changes and differences in the land use economic efficiency of urban agglomerations. According to the theoretical analysis framework and research hypotheses, this paper constructs the following panel data models:

(2) $$\begin{array}{l} \text{lnLGD}{\text{P}}_{\text{it}}={\text{β}}_{0}+{\text{β}}_{1}\text{lnI}{\text{A}}_{\text{it}}+{\text{β}}_{2}\text{lnK}{\text{L}}_{\text{it}}+{\text{β}}_{3}\text{lnT}{\text{P}}_{\text{it}}+{\text{β}}_{4}\text{lnT}{\text{C}}_{\text{it}}\\ \;+{\text{ β}}_{5}\text{lnM}{\text{I}}_{\text{it}}+{\text{β}}_{6}\text{lnT}{\text{I}}_{\text{it}}+{\text{β}}_{7}\text{lnI}{\text{I}}_{\text{it}}+\varepsilon_{\text{it}} \end{array}$$

(3) $$\begin{array}{l} \text{lnLTF}{\text{P}}_{\text{it}}={\text{β}}_{0}+{\text{β}}_{1}\text{lnI}{\text{A}}_{\text{it}}+{\text{β}}_{2}\text{lnK}{\text{L}}_{\text{it}}+{\text{β}}_{3}\text{lnT}{\text{P}}_{\text{it}}+{\text{β}}_{4}\text{lnT}{\text{C}}_{\text{it}}\\ \;+{\text{ β}}_{5}\text{lnM}{\text{I}}_{\text{it}}+{\text{β}}_{6}\text{lnT}{\text{I}}_{\text{it}}+{\text{β}}_{7}\text{lnI}{\text{I}}_{\text{it}}+\varepsilon_{\text{it}} \end{array}$$

(4) $$\begin{array}{l} \varepsilon_{it}=\mu_{i}+\lambda_{t}+u_{it} \end{array}$$

where LGDP_it_ is the land use economic productivity (single factor productivity) of the i-th urban agglomeration in the t-th year, and LTFP_it_ is the land use economic productivity (total factor productivity) of the i-th urban agglomeration in the t-th year; IA_it_ is the industrial agglomeration level of the i-th urban agglomeration in the t-th year (Krugman Specialization Index), and KL_it_ is the industrial structure change level (capital-labor ratio) of the i-th urban agglomeration in the t-th year, and TP_it_ is the number of patent application for the unit land area of the i-th urban agglomeration in the t-th year, and TC_it_ is the technical market inflow for the unit land area of the i-th urban agglomeration in the t-th year, and MI_it_ is the marketization index of the i-th urban agglomeration in the t-th year, TI_it_ is the transportation infrastructure for the unit land area of the i-th urban agglomeration in the t-th year, and II_it_ is the information infrastructure for the unit land area of the i-th urban agglomeration in the t-th year. Before the regression analysis, all the indicators were logarithmically processed. ε_it_ is the random error term, μ_i_ is the individual effect, λ_t_ is the time effect. I = 1, 2, …, 10, representing 10 urban agglomerations; t = 1, 2, …, 20, representing time 2001, 2002, …, 2020. E(μ_i_) = 0, E(λ_i_) =0, E(μ_i_u_it_) = 0, E(λ_i_u_it_) = 0.

### Index system

The index system involved in the regression equation mainly includes land use economic efficiency, industrial agglomeration, industrial structure, technological progress, marketization institution and infrastructure of urban agglomerations.

#### Land use economic efficiency of urban agglomerations

*LUEE* measurement methods are generally divided into two categories: one is the single factor productivity measurement method. Single factor productivity is an absolute efficiency indicator, and its benefit is real, easy to understand and compare. The other type is the total factor productivity measurement method. The main measurement methods include growth kernel algorithm, frontier analysis and index method. As a frontier analysis method, Data Envelopment Analysis (DEA) uses the optimization method to determine the weight of various input factors endogenously, avoiding the specific expression of the relationship between input and output, and eliminating the interference of many subjective factors on the measurement method. It also has advantages such as no relationship with market price, and is especially suitable for economic efficiency evaluation of complex economies. This paper uses both single factor productivity and total factor productivity methods, so as to more comprehensively measure *LUEE* of urban agglomerations.

First, referring to the research of scholars such as Pain (45), this paper uses the single factor productivity method to measure LUEE of urban agglomerations. LUEE is measured by the ratio of the sum of gross domestic product (GDP) to the total land area of urban agglomerations.

Second, the total factor productivity method is used to measure LUEE of urban agglomerations. This paper uses the DEA-Mamquist model to measure LUEE (total factor productivity) of urban agglomerations. The input factors are labor, capital and land, and the output factor is GDP. The capital stock is estimated using Goldsmith's Perpetual Inventory Method (PIM). The DEA-Malmquist model can be used to measure the change in total factor productivity of urban agglomerations in China from 2001 to 2020. The land use total factor productivity (LTFP) of an urban agglomeration can be expressed as:

(5) $$\begin{array}{l} L\text{TF}{\text{P}}_{\text{it}}=L\text{TF}{\text{P}}_{\text{it}-1}\times T{\text{FPCH}}_{it} \end{array}$$

Where LTFP_it_ is the land use total factor productivity of the i-th urban agglomeration in the t-th year, and TFPCH_it_ is the Malmquist index of the i-th urban agglomeration in the t-th year. This paper sets 2001 as the base period, that is, LTFP_i2001_ = 1. i = 1, 2, …, 10, representing 10 urban agglomerations; t = 1, 2, …, 20, representing time 2001, 2002, …, 2020.

#### Industrial agglomeration of urban agglomerations

There are many methods for calculating the level of industrial agglomeration. The representative methods are the structural similarity coefficient method proposed by the International Industrial Research Center of the United Nations Industrial Development Organization, the Krugman Specialization Index proposed by Paul Krugman, structural coincidence index proposed by Finger and Kreinin, and location entropy method, etc. Among them, most studies believe that the Krugman Specialization Index method performs best in measuring the level of regional industrial agglomeration. Therefore, this paper also uses the Krugman Specialization Index to measure the industrial agglomeration of urban agglomerations. The calculation formula is as follows.

(6) $$\begin{array}{l} \text{I}{\text{A}}_{\text{r}}=\frac{1}{\text{m}}\sum_{\text{i}=1}^{\text{m}}\sum_{\text{k}=1}^{\text{n}}|{\text{X}}_{\text{ik}}-{\text{X}}_{\text{k}}| \end{array}$$

Where IA_ij_ is the industrial agglomeration level of the r-th urban agglomeration (Krugman specialization index, which is the average value of the industrial agglomeration level of each city in the urban agglomeration); m is the number of cities in the urban agglomeration, k is the number of industries, and k = 1,2,3…,n; X_ik_ is the proportion of the number of employees in the k-th industry in i-th city to the number of employees in the entire industry, and X_k_ is the proportion of the number of employees in the k-th industry in all cities in the urban agglomeration to the number of employees in the entire industry in all cities.

#### Industrial structure change of urban agglomerations

The industrial structure change of urban agglomerations is measured by the capital-labor ratio (KL), which reflects the degree of the transformation of the regional industrial structure from labor-intensive industries to capital-intensive industries, so as to measure the impact of the regional industrial structure change on the land use economic efficiency.

#### Technological progress of urban agglomerations

The technological progress of urban agglomerations is measured by two indicators. One is the number of patent applications per unit of land area in the urban agglomeration (TP). That is, TP is the ratio of the total number of patent applications to the total land area of the urban agglomeration. And the other is the contract income of technical market inflow per unit of land area in the urban agglomeration (TC). That is, TC is the ratio of the total contract income of the technology market inflow to the total land area of the urban agglomeration.

#### Marketization institution of urban agglomerations

The institution is a very abstract variable, and its content and dimensions are also very rich. There are also many scholars in the academic world who are trying to measure the institution level of each urban agglomeration, and the measurement indicators used are also different. Referring to the practices of other scholars, this paper uses the marketization index in the “China Marketization Index Report” as the marketization institution variable (MI). Therefore, the marketization institution indicator score of each urban agglomeration is the arithmetic mean of the corresponding marketization index of the provinces or municipalitys included in the urban agglomeration.

#### Infrastructure of urban agglomerations

The infrastructure of an urban agglomeration is measured by two indicators. One is the level of transportation infrastructure per unit of the land area in the urban agglomeration (TI). That is, TI is the ratio of the total length of the road, railway and inland waterway of the urban agglomeration to the total land area of the urban agglomeration. The other is the level of information infrastructure per unit of the land area in the urban agglomeration (II). That is, II is the ratio of the sum of the telecom business income to the total land area of the urban agglomeration.

## Results

### The impact of the severity of the epidemic on the land market of urban agglomerations

Through the calculation of the data, the specific types of coordination between the severity of the epidemic and the degree of impact on the land market of urban agglomerations in China can be divided (Table 2).

**Table 2:** Results of coupling coefficient.

**Urban agglomerations**	**Representative** **city**	**Confirmed cases** **per million**	**Land price** **growth rate (%)**	**Coupling** **coefficient H_t_**	**Type**
Beijing-Tianjin-Hebei	Beijing	46.16	2.88	0.50	Synchronous
	Tianjin	20.23	−1.31	0.31	Strong
	Shijiazhuang	5.62	4.39	0.46	Fragile
Yangtze River Delta	Shanghai	63.34	2.62	0.49	Strong
	Hangzhou	17.47	1.91	0.51	Synchronous
	Nanjing	10.94	1.36	0.51	Synchronous
	Hefei	21.25	3.45	0.51	Synchronous
Pearl River Delta	Shenzhen	31.48	0.60	0.46	Strong
	Guangzhou	24.63	0.00	0.45	Strong
South Central of Liaoning	Shenyang	7.57	3.86	0.49	Fragile
	Dalian	22.99	2.71	0.51	Synchronous
Shandong Peninsula	Jinan	5.28	1.02	0.51	Synchronous
	Qingdao	8.32	−1.86	0.22	Strong
West coast of the strait	Fuzhou	9.23	−1.11	0.39	Strong
	Xiamen	8.16	3.69	0.49	Fragile
Central Plains	Zhengzhou	15.17	2.66	0.51	Synchronous
Middle reaches of the Yangtze River	Wuhan	4489.83	2.68	0.42	Strong
	Nanchang	41.07	2.58	0.50	Synchronous
	Changsha	28.83	2.35	0.50	Synchronous
Central Shanxi Plain	Xi'an	11.76	5.76	0.48	Fragile
Chengdu-Chongqing	Chengdu	9.53	3.44	0.50	Synchronous
	Chongqing	18.88	3.41	0.51	Synchronous

The data in the table is as of December 31, 2020.

It can be seen that the impact of the severity of the epidemic on the land market of each city in urban agglomerations shows great differences. Even within the same urban agglomeration, individual city shows differences. Hypothesis H1 passes the test. From the perspective of the coupling index, the cities where the severity of the epidemic has little impact on the land market (strong type) are all located in the eastern and central regions of China. These regions are population inflow areas with developed economies and strong support for land market demand. Cities where the severity of the epidemic and the impact on the land market are relatively consistent (synchronized type) are mainly located in the central region of China, and a few cities are located in the eastern or western regions. The impact of the land market in these cities is basically the same as the severity of the epidemic. Most of the cities where the severity of the epidemic has a greater impact on the land market (fragile type) are located in the western region of China, and a small epidemic can cause large fluctuations in the land market.

### Analysis of driving factors of land use economic efficiency

According to the theoretical hypothesis and the panel measurement model, this paper analyzes the LUEE (single factor productivity and total factor productivity) and its driving factors of urban agglomerations. In order to minimize the interference of heteroscedasticity on the regression estimation results, the regression equations use robustness estimates.

#### Stationarity test of indexs

Since many of the selected variable indexs have a time trend, in order to prevent the phenomenon of pseudo-regression, it is necessary to first test the stability of each variable index. In this paper, the four kinds of stationarity test methods of Levin-Lin-Chu panel unit root test (LLC), Im-Pesaran-Shin panel unit root test (IPS), Fisher-Augmented Dickey-Fuller test (ADF-Fisher) and Fisher-Phillips-Perron test (PP-Fisher) are used to ensure the accuracy of the test conclusion. Table 3 reflects the results of the stationarity test for each index sequence.

**Table 3:** Results of stationarity test for each index sequence.

**Index**	**LLC**	**IPS**	**Fisher-ADF**	**Fsher-PP**
lnLGDP	−0.5676 (0.2852)	4.11386 (1.0000)	10.2446 (0.9635)	2.13602 (1.0000)
Δ2lnLGDP	−5.917^***^ (0.0000)	−6.505^***^ (0.0000)	146.29^***^ (0.0000)	250.9028^***^ (0.0000)
lnLTFP	−11.2162^***^ (0.0000)	−9.044^***^ (0.0000)	116.2324^***^ (0.0000)	48.9494^**^ (0.0003)
lnIA	−1.7526^**^ (0.0398)	−1.199 (0.115)	49.2602^**^ (0.0003)	75.8893^***^ (0.0000)
lnKL	−7.856^***^ (0.0000)	−2.727^***^ (0.0000)	70.6904^***^ (0.0000)	137.3813^***^ (0.0000)
lnTP	−0.63327 (0.7246)	0.544 (0.707)	5.3956 (0.9995)	6.2283 (0.9986)
Δ2lnTP	−4.09543^***^ (0.0000)	−5.133^***^ (0.0000)	89.8465^***^ (0.0000)	331.507^***^ (0.0000)
lnTC	−0.2025 (0.4198)	0.71 (0.761)	2.5313 (1.0000)	1.9615 (1.0000)
ΔlnTC	−5.40226^***^ (0.0000)	−5.266 ^***^ (0.0000)	82.1141^***^ (0.0000)	239.7149^***^ (0.0000)
LnMI	−1.9418^**^ (0.0261)	−0.632 (0.264)	34.3275^**^ (0.0240)	31.048^*^ (0.0546)
lnTI	−1.72614^**^ (0.0422)	0.389 (0.651)	12.9433 (0.8798)	10.7979 (0.9513)
ΔlnTI	−1.41704^*^ (0.0782)	−3.373 ^***^ (0.0000)	40.4952^**^ (0.0043)	113.2694^***^ (0.0000)
lnII	−1.17004 (0.1210)	−1.082 (0.14)	16.5012 (0.6851)	21.0177 (0.3961)
ΔlnII	−4.04140^***^ (0.0000)	−4.918 ^***^ (0.0000)	83.8729^***^ (0.0000)	150.173^***^ (0.0000)

*, **, *** indicate that the index is significant at 10, 5, and 1% confidence levels, respectively.

Δ represents the first-order difference of the index, and Δ2 represents the second-order difference of the index.

According to the results of the four test statistic of each index sequence in Table 3, the original sequences of the indicators lnLTFP, lnIA, lnKL, and lnMI are stable, that is, obey the I(0) process; The original sequences of the indicators lnTC, lnTI, and lnII are not stable, but the first-order difference sequences are stable, that is, obey the I(1) process; The original sequences of the indicators lnLGDP and lnTP are not stable, but the two-order difference sequences are stable, that is, obey the I(2) process. It can be seen that the indexs are the same order. The co-integration test of the interpreted and explanatory variables can be performed before the regression analysis.

#### Cointegration test between indexs

The Pedroni cointegration test method is the most commonly used co-integration test method, which can provide multiple test statistics at the same time, thus enhancing the scientificity of the test conclusion. The co-integration test results of the interpreted variable and the explanatory variable are shown in Table 4.

**Table 4:** Co-integration test results.

***T*-Test**	**lnLGDP**	**lnLTFP**
Modified PP	4.5013^***^	4.7237^***^
PP	−1.9594^**^	−0.3431^*^
ADF	−2.2713^**^	−1.8753^**^

*, **, *** indicate that the index is significant at 10, 5, and 1%, confidence levels respectively.

From Table 4, it can be found that the Modified Phillips-Perron (Modified PP), Phillips-Perron (PP), and Augmented Dickey-Fuller (ADF) statistics of lnLGDP and lnLTFP all reject the null hypothesis that “there is no co-integration relationship.” Therefore, it can be concluded that there is a co-integration relationship between lnLGDP, lnLTFP and the variables lnIA, lnKL, lnTP, lnTC, lnMI, lnTI, lnII. Therefore, this paper can select lnLGDP, lnLTFP and lnIA, lnKL, lnTP, lnTC, lnMI, lnTI, lnII to construct panel regression model to analyze the land use economic efficiency and its driving factors of urban agglomerations.

#### Regression results

##### Regression results of LUEE (single factor productivity) of urban agglomerations

According to the research hypothesis and the econometric model, the LUEE (single factor productivity) and its driving factors of urban agglomerations (industrial agglomeration, industrial structure, technological progress, marketization institution and infrastructure) is analyzed. The regression results are shown in Table 5.

**Table 5:** Regression results of LUEE (lnLGDP, single factor productivity).

**Index**	**lnLGDP**		
	**OLS**	**FE**	**RE**
c	5.1149^***^ (0.2554)	6.5014^***^ (0.1640)	6.3709^***^ (0.1778)
lnIA	0.1945^***^ (0.0652)	0.1868^***^ (0.0448)	0.1720^***^ (0.0470)
lnKL	0.3828^***^ (0.0331)	0.4816^***^ (0.0332)	0.4619^***^ (0.0338)
lnTP	0.2762^***^ (0.0412)	0.1348^***^ (0.0243)	0.1457^***^ (0.0256)
lnTC	0.0533^*^ (0.0307)	0.0697^***^ (0.0207)	0.0638^***^ (0.0218)
lnMI	−0.3681^***^ (0. 0789)	−0. 0470 (0.0486)	−0. 0200 (0.0512)
lnTI	0.0585^*^ (0.0325)	0.2073^***^ (0.0328)	0.1959^***^ (0.0329)
lnII	0.2541^***^ (0.0345)	0.0152 (0.0300)	0.0499 (0.0308)
F Test		2392.60^***^ (0.0000)	
LM			14937.54^***^
Test			(0.000)
Hausman Test		chi^2^ < 0	
Numbers	200	200	200
*R* ^2^	0.9729	0.9892	0.9890

①*, **, *** indicate that the index is significant at 10, 5, and 1% confidence levels, respectively; ②The values in parentheses below the coefficient of each variable are the corresponding standard deviations; ③OLS, FE, and RE represent the Pooled Ordinary Least Squares Model, Fixed Effects Model, and Random Effects Model. ④The selection of the model is mainly marked by F test, Lagrangian Multiplier (LM) test and Hausman test, and the corresponding statistical value and significance level are marked.

Based on the results of the F test, LM test and Hausman test in Table 5, the fixed effect model is the optimal model of the regression equation. Analyze the estimated results of the model, this paper got some interesting findings.

From the perspective of the driving factors of LUEE (lnLGDP, single factor productivity), the statistical results of industrial agglomeration (lnIA), industrial structure change (lnKL), technological progress (lnTP, lnTC), and transportation infrastructure (lnTI) are significant and the coefficient is positive. Hypotheses H2, H3, H4, H5, and H7 pass the test, which shows that industrial agglomeration, industrial structure change, technological progress, and transportation infrastructure are conducive to the improvement of LUEE (single factor productivity) of urban agglomerations. The coefficient of marketization institution (lnMI) is negative, but the result is not statistically significant, so hypothesis H6 is not supported. The coefficient of information infrastructure (lnII) is positive but not statistically significant, so hypothesis H8 is not supported.

##### Regression results of LUEE (total factor productivity) of urban agglomerations

According to the research hypothesis and the econometric model, the LUEE (total factor productivity) and its driving factors of urban agglomerations (industrial agglomeration, industrial structure, technological progress, marketization institution and infrastructure) is analyzed. The regression results are shown in Table 6.

**Table 6:** Regression results of LUEE (lnLTFP, total factor productivity).

**Index**	**lnLTFP**		
	**OLS**	**FE**	**RE**
c	1.3464^***^ (0.3874)	1.8801^***^ (0.5004)	1.3464^***^ (0.3874)
lnIA	0.1232^***^ (0.0990)	0.0847^***^ (0.1367)	0.1232^***^ (0.0990)
lnKL	0.0401^***^ (0.0502)	0.0982^***^ (0.1015)	0.0401^***^ (0.0502)
lnTP	0.2113^***^ (0.0625)	0.1608^**^ (0.0743)	0.2113^***^ (0.0625)
lnTC	0.0859^*^ (0.0466)	0.0953 (0.0632)	0.0859^*^ (0.0466)
lnMI	−0.4916^***^ (0.1196)	−0.6447^***^ (0.1484)	−0.4916^***^ (0.1196)
lnTI	0.2530^***^ (0.0493)	0.2495^**^ (0.1000)	0.2530^***^ (0.0493)
lnII	0.0185 (0.0524)	0.1081 (0.0916)	0.0185 (0.0524)
F Test		5.11 (0.0000)	
LM Test			33.41 (0.0000)
Hausman Test			*P* > chi^2^ = 0.2318
Numbers	200	200	200
*R* ^2^	0.8172	0.8636	0.8380

The meaning of each index, model and test representative in this table is consistent with Table 5.

*, **, *** indicate that the index is significant at 10, 5, and 1% confidence levels, respectively.

It can be seen from the results of the F test, the LM test and the Hausman test in Table 6 that the random effect model is the optimal model of the regression equation. By analyzing the estimated results of the model, this paper got some interesting findings.

From the perspective of the driving factors of LUEE (lnLTFP, total factor productivity), the statistical results of industrial agglomeration (lnIA), industrial structure change (lnKL), technological progress (lnTP, lnTC), and transportation infrastructure (lnTI) are significant and the coefficient is positive. Hypotheses H2, H3, H4, H5, and H7 pass the test, which shows that industrial agglomeration, industrial structure change, technological progress, and transportation infrastructure are conducive to the improvement of LUEE (total factor productivity) of urban agglomerations. The results of these factors are consistent with tests of LUEE (single factor productivity). From a theoretical analysis, the driving mechanism behind these factors can be found. Industrial agglomeration can form economies of scale, agglomeration and cooperation, all of which are conducive to improving the land use economic efficiency of urban agglomerations. The transformation of the industrial structure from labor-intensive to capital-intensive, possibly due to technological progress, is conducive to improving the land use economic efficiency. Technological innovation activities between cities can improve the land use economic efficiency through the effects of innovation itself and spillover effects. The more developed transportation infrastructure conditions in the region (such as the developed high-speed rail network) can improve the economic connection and information exchange between the cities in the region, and it is also conducive to the availability and mobility of labor, and those directly or indirectly improve the land use economic efficiency. The coefficient of marketization institution (lnMI) is negative and statistically significant, so hypothesis H6 is not supported. This is inconsistent with the test result of LUEE (single factor productivity) (the coefficient of this indicator is negative, but the statistical result is not significant). This shows that the marketization institution is not conducive to the improvement of the LUEE (total factor productivity). The coefficient of information infrastructure (lnII) is positive, but the statistical result is not significant. Hypothesis H8 is not supported, which is consistent with the test result of LUEE (single factor productivity).

## Conclusion

This paper focuses on the evaluation of land use economic efficiency and its driving factors of urban agglomerations, and the selected samples are 10 urban agglomerations in China. To sum up, this paper draws the following main research conclusions.

First, the COVID-19 epidemic has a great impact on the land market of various cities in China's urban agglomerations, but it has shown great differences. Due to the profound changes in globalization and competition, although China's economic operation has temporarily overcome the impact of the epidemic, the foundation for economic recovery is not yet solid. The COVID-19 epidemic spreads rapidly and in many ways, posing a serious threat to human life and health. China's land system has made positive contributions to epidemic prevention and control, and played the role of safety valve, reservoir, and stabilizer. However, this epidemic has also brought challenges to China's land system, and it is urgent to propose countermeasures and suggestions to improve the land system.

Second, the empirical test of the driving factors of land use economic efficiency of in China's urban agglomerations found that some theoretical hypotheses passed the test, and some theoretical hypotheses were not supported. Industrial agglomeration, industrial structure change, technological progress, and transportation infrastructure play a significant role in promoting the land use economic efficiency of urban agglomerations. The marketization institution does not have a significant driving effect on the land use economic efficiency (single factor productivity) in urban agglomerations, nor does information infrastructure drive the land use economic efficiency (single factor productivity and total factor productivity) of urban agglomerations.

Third, whether using single factor productivity or total factor productivity, the test results of the driving factors of land use economic efficiency in China's urban agglomerations show consistency. Specifically, whether single factor productivity or total factor productivity is used to measure land use economic efficiency of urban agglomerations, the driving effects of industrial agglomeration, industrial structure change, technological progress, and transportation infrastructure are all significant. The driving effect of information infrastructure on land use economic efficiency (single factor productivity and total factor productivity) in urban agglomerations is also consistent, but the statistical result is not significant. The driving effect of the marketization institution on land use economic efficiency (single factor productivity and total factor productivity) in urban agglomerations is also consistent, but the statistical significance test is inconsistent.

According to the calculation results of land use economic efficiency in China's urban agglomerations and the analysis of driving factors, each urban agglomeration should be improved according to the decomposition of land use economic efficiency and specific driving factors. Because the driving factors are multi-dimensional, it is not only necessary to consider from a single factor, but also to analyze the total from the systematic perspective. Overall, the improvement of land use economic efficiency of China's urban agglomerations should be considered from the following aspects: First, it is necessary to take a series of measures to reform the market-oriented allocation of land elements, and improve a long-term mechanism for the smooth operation of the land market. The supply of land resources is tightening, and promoting the high-quality utilization of industrial land has become an important factor for China to stabilize investment, stabilize expectations, ensure employment, ensure supply of industrial chains, and ensure grassroots operation services. Second, on the basis of scientific assessment of the level of industrial agglomeration in urban agglomerations, strengthen industrial division and cooperation, and give better play to the role of industrial agglomeration effect, and take the prevention of congestion effect as the core content of land use management in urban agglomerations. Third, it is necessary to optimize the industrial structure of urban agglomerations, promote the rationalization and advancedization of the industrial structure, and improve the intensification level of land use. The fourth is to improve the technological innovation structure of urban agglomerations, increase investment in technological innovation, continuously improve the incentive mechanism for technological innovation, and enhance the ability of technological progress to support the sustainable development of urban agglomerations. Fifth, the combination of market mechanism and government macro-control provides an institutional basis for the high-quality development of urban agglomerations. Sixth, continue to improve the infrastructure construction of urban agglomeration, such as transportation and information communication, and use its radiation effect to promote economic interaction between cities in the urban agglomeration.

However, further research is needed as follows. First, environmental factor is not incorporated into the analytical framework of the land use economic efficiency. Future research will consider incorporating environmental and social factors into the analytical framework of land use efficiency, and analyze the coupling and coordination relationship between the land use economic efficiency, the high-quality development of the economy, and the ecological environment. The second is the spatial effects of land use economic efficiency in urban agglomerations. In the future, the spatial correlation, spatial structure characteristics and spatial effects of land use economic efficiency will be studied.

## Data availability statement

The original contributions presented in the study are included in the article/supplementary material, further inquiries can be directed to the corresponding author.

## Author contributions

The work done in the project was distributed among authors JW and JM. JW searched the back ground materials and designed the analytical characterization and empirical study and made the critical revision and editing frame. JM analyzed the data and evaluated the results. All authors have contributed to writing the paper.

## Funding

This research was financially sponsored by a grant from Major Programs of Philosophy and Social Science Research for colleges and universities in Jiangsu Province (Research on the effect evaluation of innovation policy in the National Independent Innovation Demonstration Zone in Southern Jiangsu; No. 2022SJZD065).

## Conflict of interest

The authors declare that the research was conducted in the absence of any commercial or financial relationships that could be construed as a potential conflict of interest.

## Publisher's note

All claims expressed in this article are solely those of the authors and do not necessarily represent those of their affiliated organizations, or those of the publisher, the editors and the reviewers. Any product that may be evaluated in this article, or claim that may be made by its manufacturer, is not guaranteed or endorsed by the publisher.
